# A sophisticated design of copper core to converge rotating eddy current control for detecting cracks in conductive materials

**DOI:** 10.1038/s41598-023-32319-8

**Published:** 2023-04-04

**Authors:** Le Quang Trung, Naoya Kasai, Kouichi Sekino, Seishu Miyazaki

**Affiliations:** 1 Department of Artificial Environment, Graduate School of Environment and Information Sciences, Yokohama National University, 79-5 Tokiwadai, Hodogaya, Yokohama, 240-8501 Japan; 2 Department of Mechanical and Materials Engineering, Kanagawa Institute of Industrial Science and Technology, 705-1 Shimo Imaizumi, Ebina, Kanagawa 243-0435 Japan

**Keywords:** Characterization and analytical techniques, Design, synthesis and processing, Sensors and biosensors

## Abstract

Eddy current (EC) testing has been selected as a standard candidate for detecting defects in conductive materials in the past few decades. Nevertheless, inventing EC probes capable of detecting minor defects has always been challenging for researchers due to the tradeoff between the probe dimensions and the strength of the EC generated on the surface of the test piece. Here, we use a copper core with a sophisticated design to converge the rotating EC at the tip of the copper core to detect small cracks in all directions in conductive materials. In this method, we can arbitrarily accommodate a large excitation coil so that a larger rotating uniform EC is generated in a small area of the test piece. Hence, the probe can detect cracks in all directions in conductive materials.

## Introduction

Small defects in structural components pose potential risks. Owing to the advantages of high speed, sensitivity, and efficiency^1–4^, eddy current (EC) testing is the dominant nondestructive testing applied to detect defects in conductive materials for various industries, such as the aviation^5,6^, railroad^7–9^, and petrochemical^10^ industry, and the civil engineering field, such as in steel bridges^11,12^. It is an essential method for collecting information regarding defects in the maintenance field^13,14^.

Hoshikawa et al.^15^ noted that a straight-line pattern EC induced on the test piece surface can increase the signal-to-noise ratio (SNR). This technique is known as uniform eddy current (UEC) measurement in Japan. Meanwhile, this technique is called alternating current field measurement in America and Europe. Furthermore, the realization of self-differential and self-nulling characteristics for EC probes can reduce the effect of noise signals^16,17^. The typical UEC probe was invented by Hoshikawa and is called the Hoshi probe^15,18–25^. The structure of the Hoshi probe basically consists of a tangential rectangular excitation coil and a circular or rectangular detection coil. The principle of the UEC transducer is that when a defect exists in the material, it disrupts the distribution of the UEC and alters the magnetic flux through the detection coil.

To date, many researchers continue to investigate and develop new EC probes to achieve a high SNR to predict the size of increasingly minor defects. A ferrite core was applied as the core material of the excitation coil to increase the magnetic field amplitude and thus enhance the defect detection ability owing to its high permeability^26–31^. To date, UEC probes have had a disadvantage in detecting small defects because there is a requirement for a large excitation current intensity and thus a large structure of the probe for a strong EC to be generated on the test piece surface to increase the detection sensitivity. However, this affects the small defect detection ability because the induced EC distribution is too large compared to the size of small defects. In addition, instead of traditional ECT sensors, a highly sensitive flexible eddy current array sensor is also used to detect the surface microscopic defects^32–34^. Owing to the high-frequency conductivity measurement and large excitation amperage, the magnetic field diverges around the excitation coil and covers a large area resulting in high performance for micro-defects detection. Even so, flexible eddy current array probes usually have a small number of turns of copper wire. Therefore, flat coils need conductivity measurements at high frequencies to be able to achieve good performance (usually between 100 kHz and 10 MHz). In this mode, there will be a lot of noise obviously and a shallow skin effect. Furthermore, to pursue the spatial resolution, a flexible eddy current array probe has a large spatial resolution, which will be disadvantageous when examining test pieces with a small area, especially as it cannot detect adjacent cracks. Therefore, in our previous study, an eddy current convergence (ECC) probe with a copper core having slits, hollows, and a plate fitted under the excitation coil was considered to create an extremely strong EC converging at the tip of the copper core^35,36^. Nevertheless, for the ECC probe of the previous work, the crack signal amplitude was significantly reduced when the EC lines were parallel to the crack length as compared to perpendicular to the crack length, leading to significantly affected evaluation of the crack's characteristics. A method to solve this problem is to use a pair of excitation cores with the same frequency and currents with a phase difference of 90° to generate a rotating EC on the surface of the specimen^18,19,23,37^. However, our previous study^35,36^ was hindered in creating EC rotation on the surface of the test piece because this rotation was not directly generated by the excitation coils but by the ECs converging at the tip of the copper core. In other words, to create a uniform rotating EC across the surface of the test piece, the ECs that converge at the tip of the copper core capable of uniform rotation must be controlled. Therefore, this study presents a novel rotating uniform eddy current convergence (RUECC) probe using a sophisticated design of a copper core that can create rotating ECs converging at the tip of the copper core, resulting in the generation of an extremely strong rotating EC on the test piece surface to detect small cracks in all directions. By adjusting the size and number of turns of the excitation coil and the structure of the copper core to produce ECs that converge at the tip of the copper core, the RUECC probe can overcome the disadvantage of ECC probes in the previous studies^35,36^. The ability to detect small defects in all directions with the probe is expected to significantly improve too. Besides, a circular detection coil adhering to self-nulling and self-differential characteristics and resulting in the RUECC probe eliminating noise signals (especially noise signals during lifting). The finite element analysis was proformed to confirm the convergence of the eddy currents at the tip of the copper core. This study successfully obtained the RUECC at the tip of the copper core by manufacturing the copper core based on the finite element analysis results. Excellent defect detection ability was obtained with the special design of the copper core in comparison to the existing literature.

### Structure of the RUECC probe

**Figure 1:**
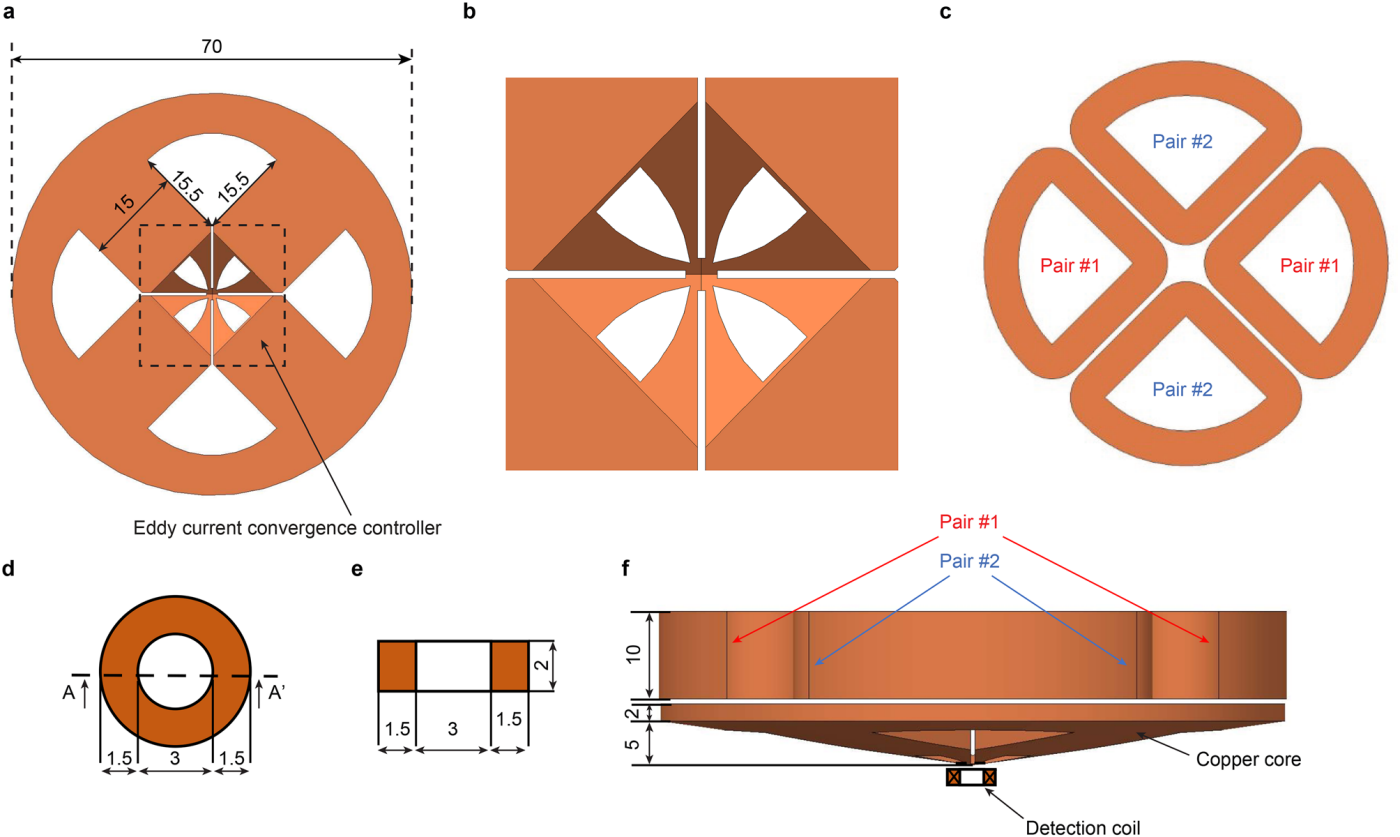
Structure of the RUECC probe (unit in mm). (**a**) Copper core. (**b**) EC convergence controller. (**c**) Two pairs of double excitation coils. (**d**,**e**) Top view and section view along A-A′ of the detection coil. (**f**) Overall RUECC probe.

The structures of the RUECC probe components are shown in Fig. 1. The sophisticated design of the copper core that can create rotating ECs converging at the tip of the copper core is shown in Fig. 1a,b. Two pairs of double excitation coils with the same plane and dimensions (Fig. 1c) were placed above the copper core (Fig. 1f) so that the ECs converging at the tip of the copper core induced by the two pairs of double excitation coils were rotated at equal amplitude in all directions. The number of turns in each excitation coil was 1000, with a copper wire 0.2 mm in diameter. The dimensions of the circular detection coil are shown in Fig. 1d,e, which was placed at the bottom and center of the copper core (Fig. 1f). The number of turns in the detection coil was 854, with a copper wire 0.05 mm in diameter.

### Authenticating the small crack detection ability of the RUECC probe

**Figure 2:**
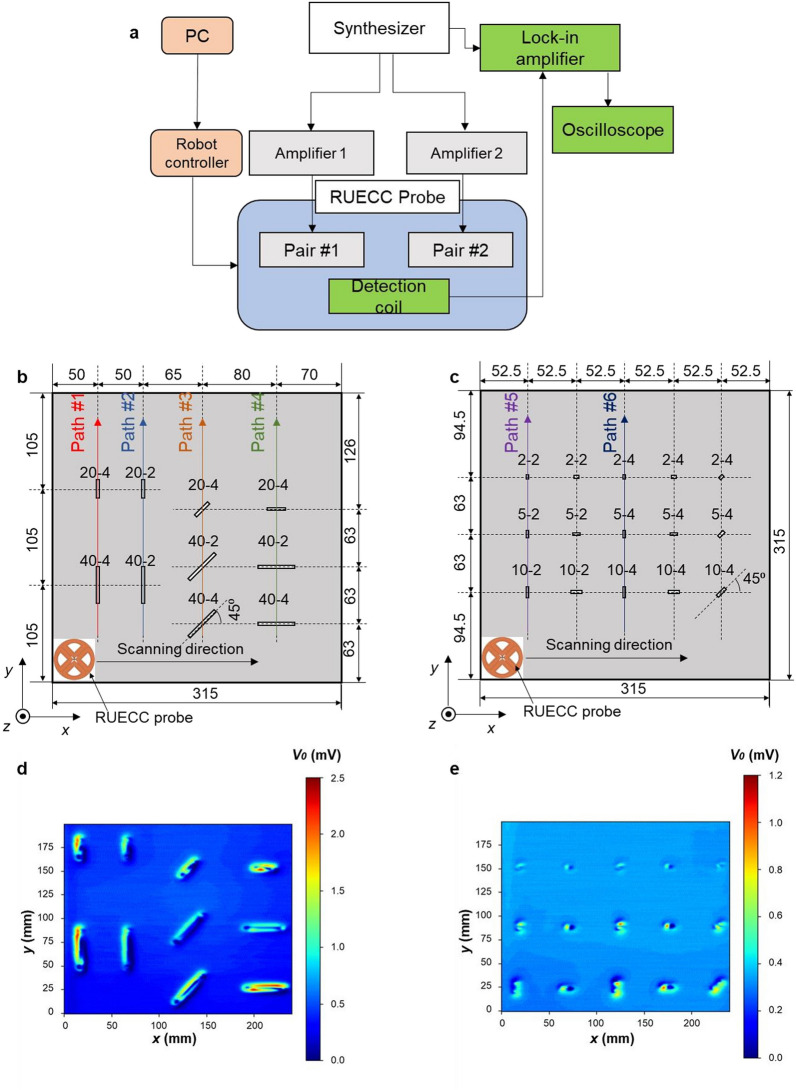
Experimental implementation with the RUECC probe. (**a**) Diagram of the experimental setup. (**b**) Experimental implementation on test piece 1. (**c**) Experimental implementation on test piece 2. (**d**) Experimental results of test piece 1. (**e**) Experimental results of test piece 2.

The experimental setup is shown in Fig. 2a. The two sine wave excitation currents with a phase difference of 90° had a frequency and a magnitude of 10 kHz and 10 mA, respectively, and these were generated using a function generator (WAVE FACTORY WF1946B, NF Co., Yokohama, Japan) and two high-speed bipolar amplifiers (NF HAS 4012, NF Co., Yokohama, Japan). A computer-controlled positioning robot module was used to move the RUECC probe over the scanning surface of the test piece at a speed of 10 mm/s. In the scanning direction, the RUECC probe was dragged along the x-axis, and this process was repeated by shifting the probe along the y-axis. Two 5052 aluminum plates with cracks of different dimensions (L-D symbol: L: length of the crack (mm); D: depth of the crack (mm); all the crack widths were 0.5 mm) and orientations (Fig. 2b,c) were prepared as test pieces to validate the ability to detect the cracks based on the method involving rotating ECs converging at the tip of the copper core. The experiments were carried out on test specimen 1 having cracks with lengths of 40 and 20 mm and depths of 2 and 4 mm (Fig. 2b) to evaluate the performance of the RUECC probe and to evaluate the self-nulling and self-differential properties in the noise signal rejection. Furthermore, the experiment was conducted on test specimen 2 (Fig. 2c) with small cracks located near each other to evaluate the performance of the RUECC probe for small crack detection. The artificial cracks were created by electrical discharge machining. The scanning interval was 1 mm in both *x* and *y* directions. The output amplitude signals were obtained by the single detection coil, processed with a two-phase lock-in amplifier (NF 5601B) and stored in a digital oscilloscope (Graphtec GL7000).

The experimental results obtained with the RUECC probe using test pieces 1 and 2 are shown in Fig. 2d,e, respectively. In general, the measurement results clearly discriminate the signals from cracks with different dimensions and orientations. However, there was a small effect on the crack signal amplitude of the cracks inclined at 45° (along Path # 3) from the x-axis as compared to that of the cracks with another orientation. It can be clearly seen that the peak signal magnitudes of 40-4, 40-2, and 20-4 along Paths # 1, # 2, and # 4 were similar while those along Path # 3 were reduced. This occurred because adjusting the output signal amplitude to zero mV (approximately 0.3 mV in experimental implementation) when there is no crack under the RUECC probe was extremely difficult in experimental implementation utilizing a manual method due to the smaller area of the strongly induced EC and the smaller size of the circular detection coil. In addition, RUECs are not same amplitude in all direction, since the RUECC in 45° is the composition of RUECs at 0 and 90°. Resultantly, the EMFs of the detection coil will suffer a small effect when the cracks were inclined 45° as compared to the parallel or perpendicular scanning directions. This effect is demonstrated through the difference in the amplitude of the two peaks of the crack signal (Fig. 2d). Therefore, the RUECC probe must have self-nulling and self-differential characteristics^16,17^ to enhance the sensitivity to the highest degree and to accurately assess the physical properties of cracks, especially for small cracks. However, two crack signal peaks were evident for the small cracks in specimen 2 (Fig. 2e) because the small disruption of large EC intensity in the surface of the test specimen resulted in negligible effect on the small crack signals. This represents the extreme sensitivity of the RUECC probe in detecting small cracks.

### Validating the crack detection ability of the RUECC probe through six paths

**Figure 3:**
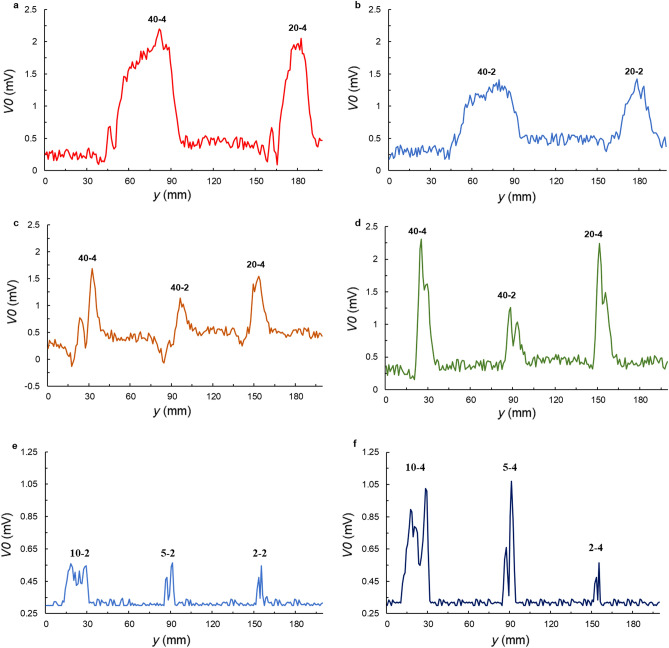
Measurement results of six paths obtained with the RUECC probe. (**a**) Path #1 (the length of cracks 4 mm deep in test specimen 1 was perpendicular to the *x*-axis). (**b**) Path #2 (the length of cracks 2 mm deep in test specimen 1 was perpendicular to the *x*-axis). (**c**) Path #3 (the crack length in test specimen 1 was inclined 45° from the x-axis). (**d**) Path #4 (the crack length in test specimen 1 was parallel to the *x*-axis. (**e**) Path #5 (the length of cracks 2 mm deep in test specimen 2 was perpendicular to the *x*-axis). (**f**) Path #6 (the length of cracks 4 mm deep in test specimen 2 was perpendicular to the *x*-axis).

To validate the crack detection ability of the RUECC probe in all directions to authenticate the rotating EC induced on the surface of the test piece by the RUECC probe, the measurement results of the RUECC probe in test piece 1 along Path #1 (the length of cracks 4 mm deep was perpendicular to the x-axis), Path #2 (the length of cracks 2 mm deep was perpendicular to the x-axis), Path #3 (the crack length was inclined 45° from the x-axis), and Path #4 (the crack length was parallel to the x-axis) were obtained, as shown in Fig. 3a–d, respectively. In addition, the measurement results of the RUECC probe in test piece 2 along Path #5 (the length of cracks 2 mm deep was perpendicular to the *x*-axis) and Path #6 (the length of cracks 4 mm deep in test specimen 2 was perpendicular to the *x*-axis) were obtained, as shown in Fig. 3e,f, respectively.

The overview measurement results show that the RUECC probe successfully detected all cracks in two test specimens. Based on the measurement result of paths #1 to #4 in test piece 1, It can be seen that the peak signal amplitude of cracks with a depth of 4 mm (40-4, 20-4) was twice that of cracks with a depth of 2 mm (40-2, 20-2). Similarly, the maximum signal amplitude of cracks with a depth of 4 mm (10-4, 5-4) was twice to that of cracks with a depth of 2 mm (10-2, 5-2) was obtained by the measurement results of Paths #5 and #6 in test piece 2. For cracks with length 2 mm, since UECs was easier to divert cracks than crawling under cracks, the maximum signal amplitude was same between cracks with length 2 mm and depths 2 mm and 4 mm. This exhibits that the peak crack signal amplitude represents the crack depth. Moreover, the distance between two corner edges of the crack signal in test specimen 1 for a crack with a length of 40 mm (40-4, 40-2) was twice that for a crack with a length of 20 mm (20-4, 20-2). Likewise, the distance between the two corner edges of the signal crack for a crack in test piece 2 with a length of 10 mm (10-4, 20-2) is double of a crack with a length of 5 mm (5-4, 5-2). Hence, the distance between the two corner edges of the crack signal reflects the crack length. In addition, the distances between the two corner edges of the crack signals obtained for Path #4 (perpendicular to the crack length) were similar because all cracks had the same width of 0.5 mm. Therefore, the distance between two corner edges of the crack signal indicates the width of the crack when the obtained measurement results are perpendicular to the crack length. Although the output signal amplitude to 0.3 mV (the ideal condition is zero according to the self-nulling and self-differential characteristics) when there is no crack under the RUECC probe, it is easy to distinguish the crack signal (especially the small crack in Fig. 2e) when the measured signal changes from a finite baseline, which is 0.3 mV as represented in Fig. 3.

Based on the measurement results of Path #3 and Path #4, the two peaks of the crack signals were not similar (Fig. 3c,d). The crack signal amplitudes of Path #3 (Fig. 3c) when the crack length was inclined 45° from the x-axis were little reduced compared to those of the other paths. This occurs because the output signal amplitude is approximately 0.3 mV when there is no crack under the RUECC probe, leading to an effect on the balanced condition^16^. This demonstrates that self-nulling and self-differential characteristics^16,17^ are essential for the RUECC probe to enhance the crack detection ability. The crack detection ability is slightly affected when the crack length is approximately 45° from the x-axis and is not affected for crack lengths in the other directions. This eliminates the most significant disadvantage of previous studies^35,36^. In addition, note that the distance between two corner edges of the crack signal obtained for Path #3 was not like the that obtained for the other paths because the measurement result was inclined 45° from the crack length or width. In other words, the distance between two corner edges of the crack signal obtained for Path #3 does not judge the length or width of the crack.

Considering the crack signal in test piece 2 based on the measurement results of Paths #5 and #6, the peak signal amplitude of cracks with a depth of 2 mm (10-2, 5-2, 2-2) are relatively similar, as shown in Fig. 3e. However, there was a marked reduction in the peak signal amplitude of the crack with a depth of 4 mm and length 2 mm (2-4) as compared to that of lengths 10 mm and 5 mm (10-4, 5-4), as shown in Fig. 3f. This indicates that the RUEC intensity generated in the test piece is not large enough to detect defects less than defects 2-4. In this case, we can adjust the excitation current magnitude as well as the frequency of the excitation coils to enhance the RUEC intensity generated in the test specimen, as reported in Ref.^36^.

## Conclusions

According to the measurement results, although there was a small effect on the results obtained with the RUECC probe when the crack length was approximately 45° from the* x*-axis, the probe had excellent sensitivity in its ability to detect cracks of different dimensions in all directions. This authenticated the ability to create strong converging ECs rotating at the tip of the copper core, leading to the generation of an extremely strong rotating EC on the surface of the test piece. Owing to this method, controlling the ECs generated on the test piece at the magnitude and direction to detect small cracks by adjusting the size and number of turns of the excitation coil and the structure of the copper core is straightforward. As a result, the RUECC probe can overcome the disadvantages of ECC probes in previous studies^32,33^ and possess the ability to detect small defects in all the directions with the probe with significant improvement.

## Methods

### Method of generating an RUEC on the surface of the test piece

To authenticate the ability of ECs to converge at the tip of the copper core through the sophisticated design of the copper core and generate an RUEC on the test piece surface, the RUECC probe model with the same dimensions as in Fig. 1 was simulated using Magnet software (version 7.9.0.18, Mentor Graphics Corporation) using time-harmonic 3D analysis.

**Figure 4:**
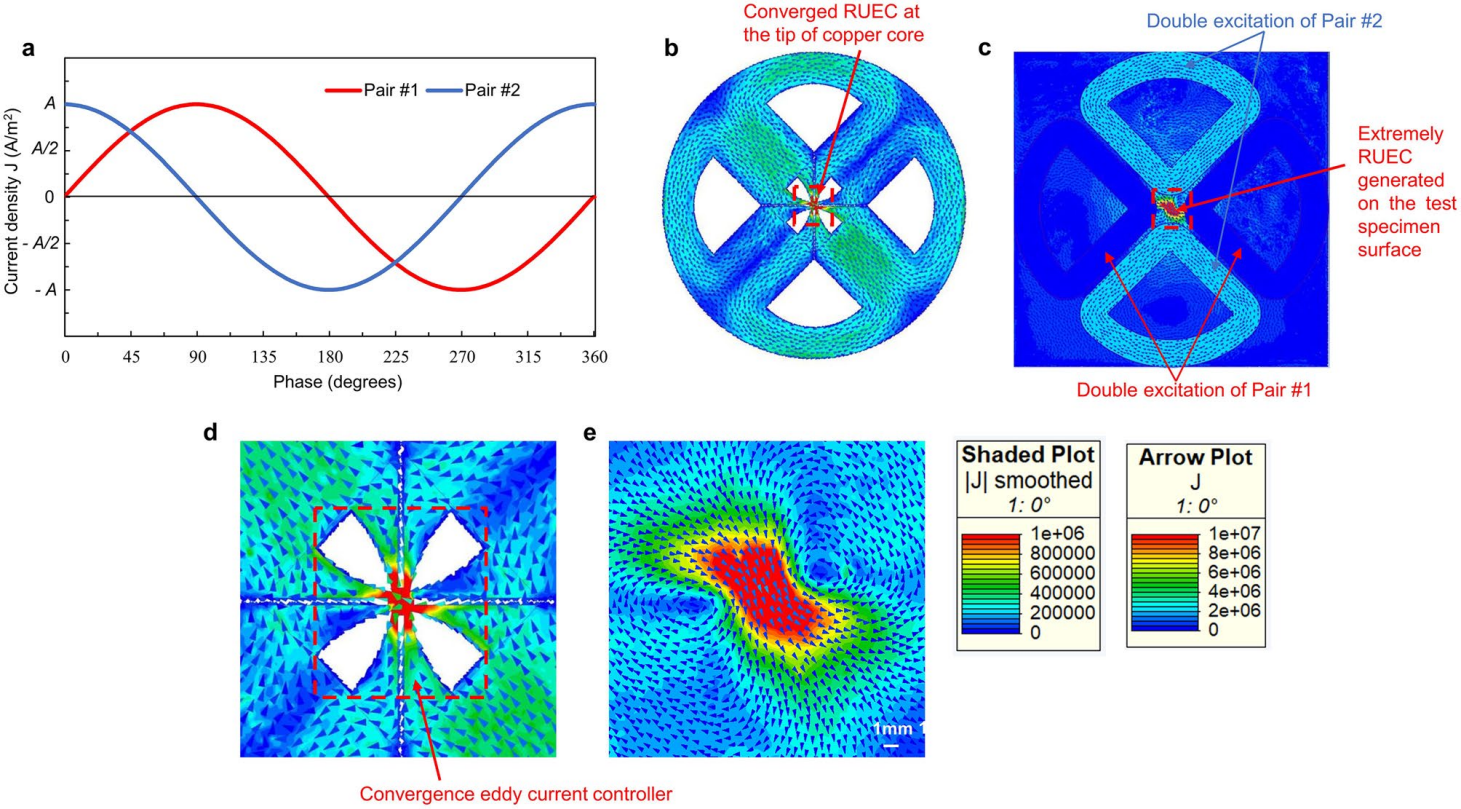
Principle of generating an RUEC with the RUECC probe. (**a**) Graph of two alternating current sources 90° out of phase with each other. (**b**) ECs converging at the tip of the copper core. (**c**) Contour and arrow plots of the EC distribution on the excitation coil and RUEC generated on the test piece. (**d**) EC convergence controller. (**e**) Contour and arrow plots of the RUEC distribution on the test specimen.

Figure 4 indicates the principle of the RUEC generated on the test piece surface owing to the ECs converging at the tip of the copper core. In this method, two alternating excitation current sources with a phase difference of 90° were applied for the two pairs of double excitation coils (Fig. 4a). Because the phase of Pair #2 is 90° earlier than that of Pair #1, the ECs induced on the copper core by Pair #1 and Pair #2 are:

1 $${EC}_{1}=Acos(\omega t+\varphi )$$

2 $${EC}_{2}=Acos\left(\omega t+\varphi +\frac{\pi }{2}\right)=Asin(\omega t+\varphi )$$

Therefore, the total EC induced on the copper core is calculated by the equation:

3 $${EC}_{total}=\sqrt{{({EC}_{1})}^{2}+{({EC}_{2})}^{2}}=\sqrt{{[Acos\left(\omega t+\varphi \right)]}^{2}+{[Asin\left(\omega t+\varphi \right)]}^{2}}=\left|A\right|$$

where ${EC}_{total}$ is the total EC generated on the copper core and ${EC}_{1}$ and ${EC}_{2}$ are the ECs generated on the copper core by double excitation coil Pairs #1 and #2, respectively. $A$ and $\varphi$ are the amplitude of current density and phase, respectively, and $\omega =2\pi f=2\pi /T$, with $f$ and $T$ being the frequency and period, respectively. Equation (3) shows that the total EC induced on the copper core constantly rotates with period *T* = 1*/f* and constant amplitude.

Figure 4b shows the contour and arrow plots of the EC distribution on the copper core of EC convergence probe. Moreover, the contour and arrow plots of the EC distribution on the excitation coil and RUEC generated on the test piece are shown in Fig. 4c. Besides, the contour and arrow plots of the RUEC, distribution on the test specimen also are shown in Fig. 4e.

Consider a particular case at time t = 0 when the amplitude of Pair #1 is zero while that of Pair #2 reaches its maximum value (Fig. 4a,c). Owing to the sophisticated design of the copper core, the induced rotating ECs converge at the tip of the copper core (Fig. 4b,d), resulting in the generation of an extremely strong RUEC on the test piece surface (Fig. 4c,e). Figure 4c demonstrates that the generated RUEC has a significantly higher amplitude than the excitation current applied to the excitation coils.

### 3D-finite element simulation results

**Figure 5:**
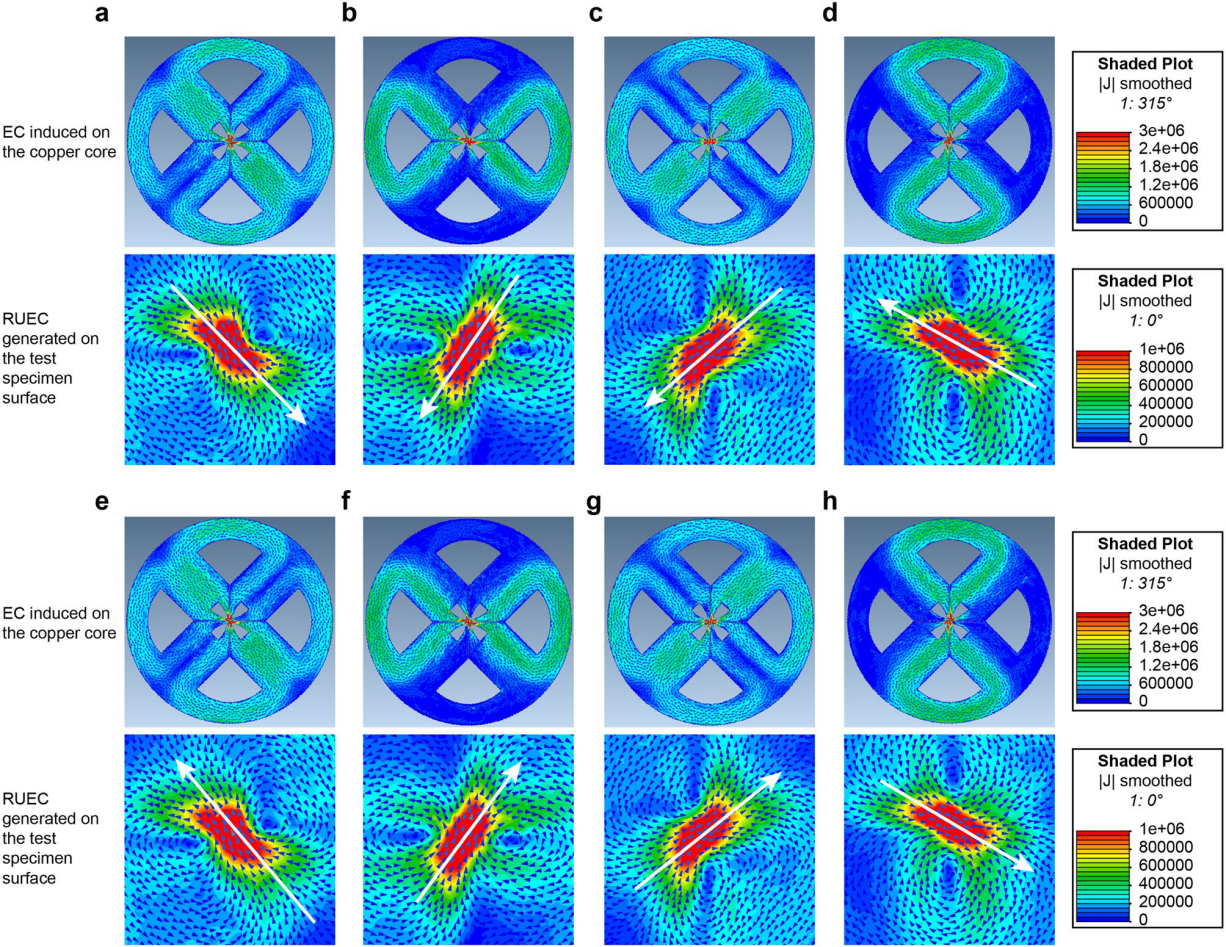
Simulation results of RUEC distribution patterns with the RUECC probe. (**a**) At 0°. (**b**) At 45° (π/4). (**c**) At 90° (π/2). (**d**) At 135° (3π/4). (**e**) At 180° (π). (**f**) At 225° (5π/4). (**g**) At 270° (3π/2). (**h**) At 315° (7π/4).

Figure 5 shows the contour and arrow plots of the EC distribution on the surfaces of the copper core and test piece. According to the simulation results, the converging ECs rotate at the tip of the copper core, leading to the generation of an RUEC on the test piece surface. When the phases were 0 and 180°, the amplitude of Pair #1 was zero while that of Pair #2 was maximum but of opposite polarity (Fig. 4a). As a result, the UECs generated on the test piece surface had opposite directions (Fig. 5a,e). In contrast, when the phases were 90 and 270°, the amplitude of Pair #2 was zero while that of Pair #1 was maximum but of opposite polarity (Fig. 4a). Consequently, the UECs generated on the test piece surface had opposite directions (Fig. 5c,e). The same occurred for the phases of 45 and 225° (Fig. 5b,f) and 135 and 315° (Fig. 5d,h). Thus, the constant-intensity converging UEC produced on the test piece surface constantly rotated with period *T.* Hence, the EMF generated on the circular detection coil also rotated, providing the ability to detect cracks in all directions.

### Principle of the output detection signal

**Figure 6:**
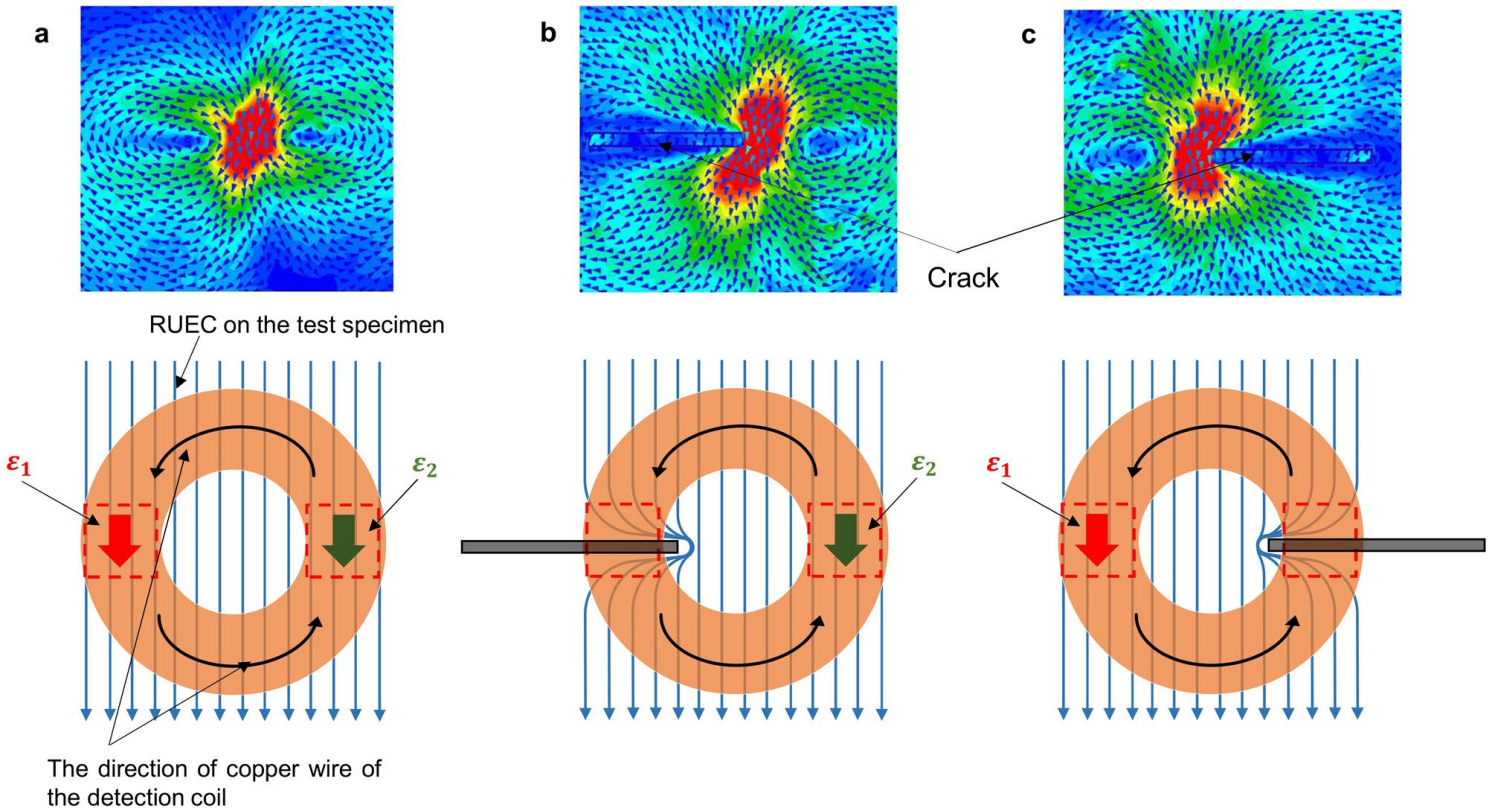
Principle of the output detection signal with the circular detection coil. (**a**) Without a crack. (**b**) With a crack under the left side of the detection coil. (**c**) With a crack under the right side of the detection coil.

Figure 6 explains the principle of the output detection signal of the RUECC probe. The principle is the same as that in Refs.^16,17^. The output detection signal depends on the EMFs generated on the detection coil by the RUEC. The EMFs are generated in the interaction zone (the red dotted line) when the direction of the copper wire of the detection coil is parallel to the RUEC. There are two conditions: the balanced condition and unbalanced condition.

The balanced condition occurs when EMFs ${\varepsilon }_{1}$ and ${\varepsilon }_{2}$ generated in the detection coil are of equal intensity but opposite polarities (Fig. 6a), leading to them cancelling each other out, called the self-differential characteristic. As a result, the output detection signal is zero, called the self-nulling characteristic. Meanwhile, the unbalanced condition occurs when there is a crack under the detection coil of the RUECC probe, causing the intensity value of ${\varepsilon }_{1}$ (Fig. 6b) or ${\varepsilon }_{2}$ (Fig. 6c) to be altered due to the disruption of the RUEC caused by the crack. Therefore, the self-nulling nature is broken, resulting in the generation of the crack detection signal.

## Supplementary Information

Supplementary Information 1.

Supplementary Video 1.

Supplementary Video 2.

Supplementary Video 3.

## Data Availability

Data generated or analysed during this study are included in the Supplementary Information. The data used in this current study are available from the corresponding authors at a reasonable request.
