# Supplementary material for: A sophisticated design of copper core to converge rotating eddy current control for detecting cracks in conductive materials
Source: Sci Rep. 2023 Apr 4;13:5479. doi: 10.1038/s41598-023-32319-8 (PMC10073206; doi:10.1038/s41598-023-32319-8)
Supplement: Supplementary file 1 — Supplementary Information 1. [file 41598_2023_32319_MOESM1_ESM.docx]

**Supplementary information**

**Supplementary Videos Legends**

**Supplementary video 1:** Contour and arrow plots of the EC distribution on the copper core of EC convergence probe.

**Supplementary video 2:** Contour and arrow plots of the EC distribution on the excitation coil and test piece.

**Supplementary video 3:** Contour and arrow plots of the EC distribution on the test piece.

The electromagnetic parameters used in this analysis are listed in Supplementary Table 1. An aluminum plate with dimensions of 70 × 70 × 10 mm was used as the test piece. The excitation current and frequency of the RUECC probe were 10 mA and 10 kHz, respectively.

**Supplementary Table 1.** Electromagnetic parameters used in the analysis.

|  | Material | Electrical conductivity, *σ*  (MS/m) | Relative permeability, *μ*_r_ |
| --- | --- | --- | --- |
| Wire of excitation coil | Copper | 57.7 | 1.0 |
| Wire of detection coil |  |  |  |
| Core of coil |  |  |  |
| Test piece | Aluminum | 38.0 |  |

All artificial cracks on the test piece were made by electrical discharge machining. Fig. 2b shows relatively large cracks, whereas Fig. 2c shows relatively small cracks. The dimensions of each crack are presented in Supplementary Table 2.

**Supplementary Table 2.** Sizes of four artificial cracks in the aluminum plate.

| Test piece | Symbol of crack | Width (mm) | Length (mm) | Depth (mm) |
| --- | --- | --- | --- | --- |
| 1 | 20-2 | 0.5 | 20 | 2 |
|  | 20-4 |  |  | 4 |
|  | 40-2 |  | 40 | 2 |
|  | 40-4 |  |  | 4 |
|  | 2-2 |  | 2 | 2 |
| 2 | 2-4 |  |  | 4 |
|  | 5-2 |  | 5 | 2 |
|  | 5-4 |  |  | 4 |
|  | 10-2 |  | 10 | 2 |
|  | 10-4 |  |  | 4 |

The specifications of each excitation coil and the circular detection coil for the RUECC probe are listed in Supplementary Table 3.

**Supplementary Table 3.** Specifications of each excitation coil and the circular detection coil for the RUECC probe.

| Each excitation coil | Turns | 1000 |
| --- | --- | --- |
|  | Wire diameter　(mm) | 0.2 |
|  | Resistance　(Ω) | 38 |
|  | Impedance　(Ω) | 104 |
|  | Inductance　(mH) | 15 |
| Detection coil | Turns | 854 |
|  | Wire diameter　(mm) | 0.05 |
|  | Resistance　(Ω) | 103 |
|  | Impedance　(Ω) | 105 |
|  | Inductance　(mH) | 2.5 |

The experimental implementation is shown in Supplementary Fig. 1. The RUECC probe was moved by using a positioning robot controller (Supplementary Fig. 1a and b). The actual shapes of the copper core, excitation coils and circular detection coil are shown in Supplementary Fig. 1d, e and f, respectively.


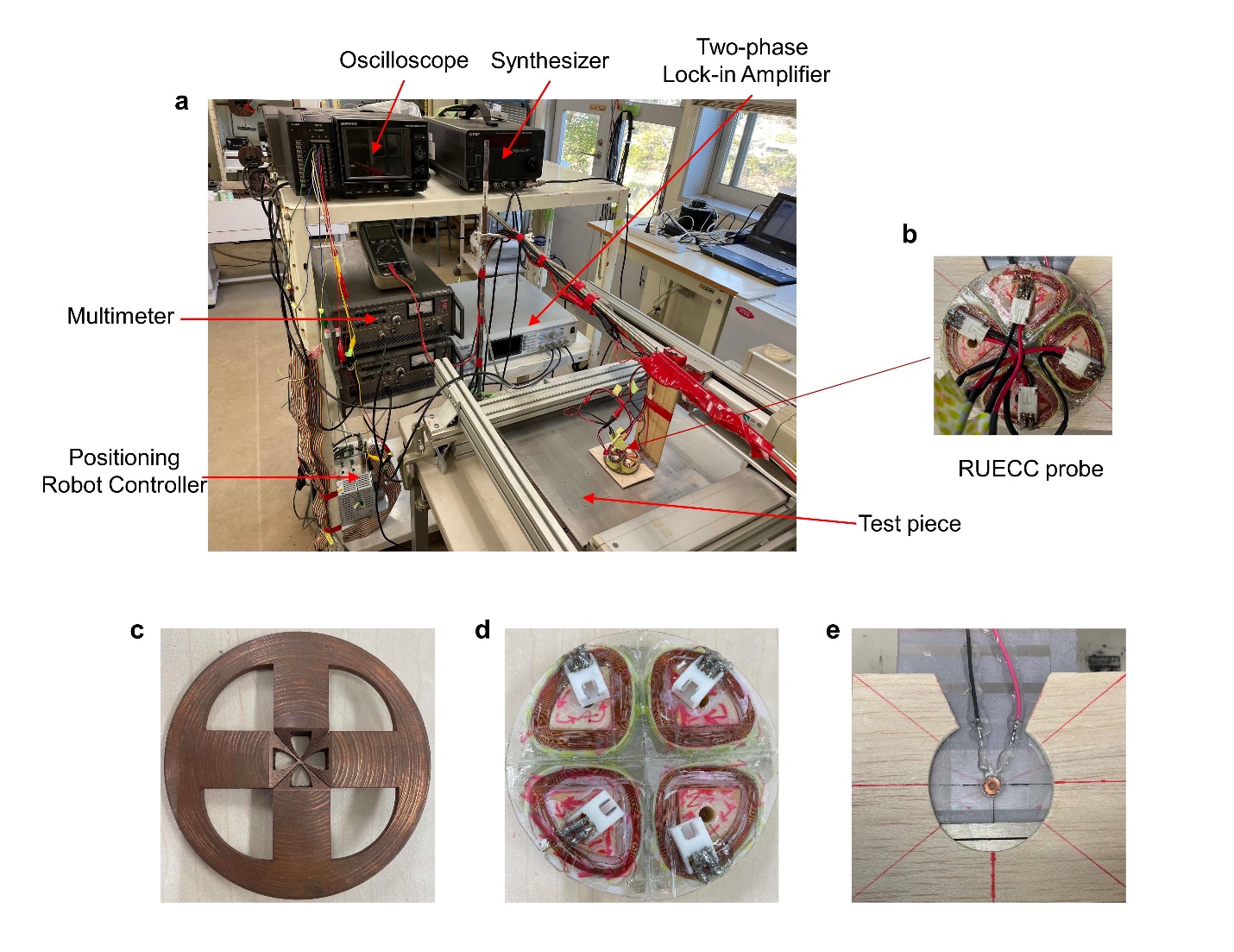


**Supplementary Fig. 1.** **Experimental implementation. a,** Experimental setup. **b,** RUECC probe. **c,** Actual shape of the copper core. **d,** Actual shape of the excitation coils. **e,** Actual shape of the circular detection coil.
